# Bis­(2-amino-5-methyl­pyridinium) fumarate–fumaric acid (1/1)

**DOI:** 10.1107/S1600536810027960

**Published:** 2010-07-24

**Authors:** Madhukar Hemamalini, Hoong-Kun Fun

**Affiliations:** a X-ray Crystallography Unit, School of Physics, Universiti Sains Malaysia, 11800 USM, Penang, Malaysia

## Abstract

In the crystal structure of the title compound, C_6_H_9_N_2_^+^·0.5C_4_H_2_O_4_^2−^·0.5C_4_H_6_O_4_, the fumarate dianion and fumaric acid mol­ecule are located on inversion centres. The 2-amino-5-methyl­pyrimidinium cation inter­acts with the carboxyl­ate group of the fumarate anion through a pair of N—H⋯O hydrogen bonds, forming an *R*_2_^2^(8) ring motif. These motifs are centrosymmetrically paired *via* N—H⋯O hydrogen bonds, forming a complementary *DDAA* array. The carboxyl groups of the fumaric acid mol­ecules and the carboxyl­ate groups of the fumarate anions are hydrogen bonded through O—H⋯O hydrogen bonds, leading to a supra­molecular chain along [101]. The crystal structure is further stabilized by weak C—H⋯O hydrogen bonds.

## Related literature

For details of fumaric acid, see: Batchelor *et al.* (2000 ▶). For related structures, see: Hemamalini & Fun (2010**a* ▶,*b* ▶,c* ▶); Nahringbauer & Kvick (1977 ▶). For hydrogen-bond motifs, see: Bernstein *et al.* (1995 ▶). For *DDAA* arrays, see: Robert *et al.* (2001 ▶); Umadevi *et al.* (2002 ▶); Thanigaimani *et al.* (2007 ▶). For carbox­yl–carboxyl­ate inter­actions, see: Büyükgüngör & Odabaşoğlu (2002 ▶); Büyükgüngör *et al.* (2004 ▶). For bond-length data, see: Allen *et al.* (1987 ▶). For the stability of the temperature controller used in the data collection, see: Cosier & Glazer (1986 ▶).
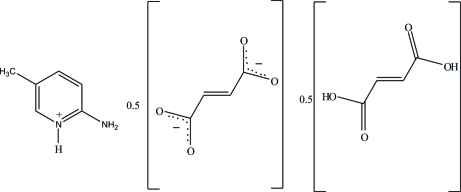


## Experimental

### 

#### Crystal data

C_6_H_9_N_2_^+^·0.5C_4_H_4_O_4_^2−^·0.5C_4_H_2_O_4_
*M* *_r_* = 224.22
Triclinic, 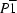

*a* = 4.0366 (4) Å
*b* = 9.3145 (10) Å
*c* = 14.0077 (14) Å
α = 94.030 (3)°
β = 95.060 (3)°
γ = 90.903 (3)°
*V* = 523.20 (9) Å^3^
*Z* = 2
Mo *K*α radiation
μ = 0.11 mm^−1^
*T* = 100 K
0.61 × 0.22 × 0.20 mm

#### Data collection

Bruker APEXII DUO CCD area-detector diffractometer
Absorption correction: multi-scan (*SADABS*; Bruker, 2009 ▶) *T*_min_ = 0.935, *T*_max_ = 0.978
19772 measured reflections
5445 independent reflections
4852 reflections with *I* > 2σ(*I*)
*R*_int_ = 0.019

#### Refinement

*R*[*F* ^2^ > 2σ(*F* ^2^)] = 0.036
*wR*(*F* ^2^) = 0.110
*S* = 1.05
5445 reflections
147 parameters
H-atom parameters constrained
Δρ_max_ = 0.60 e Å^−3^
Δρ_min_ = −0.34 e Å^−3^

### 

Data collection: *APEX2* (Bruker, 2009 ▶); cell refinement: *SAINT* (Bruker, 2009 ▶); data reduction: *SAINT*; program(s) used to solve structure: *SHELXTL* (Sheldrick, 2008 ▶); program(s) used to refine structure: *SHELXTL*; molecular graphics: *SHELXTL*; software used to prepare material for publication: *SHELXTL* and *PLATON* (Spek, 2009 ▶).

## Supplementary Material

Crystal structure: contains datablocks global, I. DOI: 10.1107/S1600536810027960/is2576sup1.cif

Structure factors: contains datablocks I. DOI: 10.1107/S1600536810027960/is2576Isup2.hkl

Additional supplementary materials:  crystallographic information; 3D view; checkCIF report

## Figures and Tables

**Table 1:** Hydrogen-bond geometry (Å, °)

*D*—H⋯*A*	*D*—H	H⋯*A*	*D*⋯*A*	*D*—H⋯*A*
N1—H1⋯O4	0.86	1.89	2.7305 (7)	167
N2—H2*A*⋯O3	0.86	1.98	2.8334 (7)	175
N2—H2*B*⋯O3^i^	0.86	2.04	2.8329 (7)	154
O2—H2*C*⋯O4	0.82	1.75	2.5618 (7)	170
C5—H5⋯O1^ii^	0.93	2.46	3.3582 (9)	162

Symmetry codes: (i) 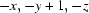; (ii) 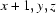.

**Fig. 1.**
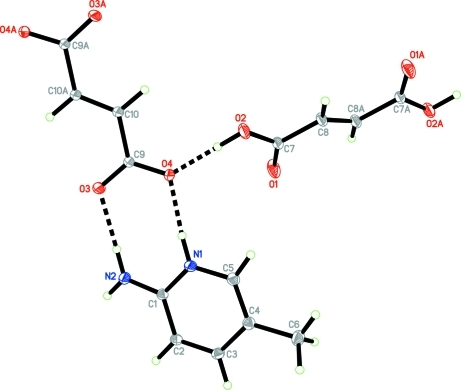
The title compound with displacement ellipsoids drawn at the 50% probability level. O1A/O2A/C7A/C8A and O3A/O4A/C9A/C10A are generated by the symmetry codes -x + 1, -y, -z and -x, -y, -z, respectively.

**Fig. 2.**
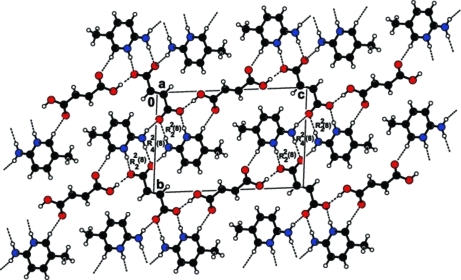
The DDAA hydrogen-bonding pattern in (I). Dashed lines indicate hydrogen bonds. These 2D patterns are stacked along the a-axis.

**Fig. 3.**
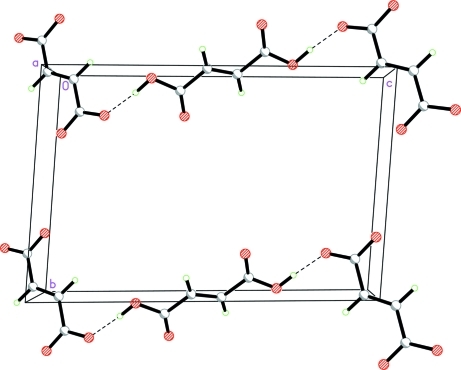
The carboxyl-carboxylate interactions of the title compound (I), viewed down the a axis, forming a 1D supramolecular chain along [101].

## supplementary crystallographic information

### Comment

Recently we have reported the crystal structures of 2-amino-5-methylpyridinium 4-nitrobenzoate (Hemamalini & Fun, 2010*a*), 2-amino-5-methylpyridinium 3-aminobenzoate (Hemamalini & Fun, 2010*c*). and 2-amino-5-methylpyridinium nicotinate (Hemamalini & Fun, 2010*b*). Fumaric acid is of interest since it is known to form supramolecular assemblies with *N*-aromatic complexes (Batchelor *et al.*, 2000). Herein we report the crystal structure and supramolecular patterns of the new compound containing pyridine derivative and fumaric acid components.

The title compound (I) is shown in Fig. 1. The asymmetric unit contains one 2-amino-5-methylpyridinium cation, a half of the fumarate anion and a half of the fumaric acid molecule. The dihedral angles between pyridinium ring and the planes formed by the fumarate anion and fumaric acid molecule are 10.53 (2)° and 55.21 (2)°, respectively. The planar fumarate and fumaric acid molecule is centrosymmetric with the mid-point of the C═C double bond located at an inversion center. In the fumaric acid, the C7—O1 bond distance of 1.2118 (7) Å is much shorter than the C7—O2 bond distance of 1.3199 (7) Å suggesting that the carboxyl group is not deprotonated in the crystal structure.  The 2-amino-5-methyl pyridinium cation is essentially planar with a maximum deviation of 0.011 (1) Å for atom N1. The 2-amino-5-methylpyridine is protonated at N1 which is evident from the increase in the internal angle at N1 (C1—N1—C5) from 117.4 (3)° in neutral 2-amino-5-methylpyridine (Nahringbauer & Kvick, 1977) to 123.03 (5)° in the present study.  The bond lengths (Allen *et al.*, 1987) and angles are within normal ranges.

In the crystal packing (Fig. 2), the protonated N1 atom and the 2-amino group (N2) is hydrogen-bonded to the carboxylate oxygen atoms (O3 and O4) via a pair of intermolecular N1—H1···O4 and N2—H2A···O3 hydrogen bonds forming a ring motif *R*_2_^2^(8) (Bernstein *et al.*, 1995). These motifs are centrosymmetrically paired via N2—H2B···O3 hydrogen bonds to produce the *DDAA* (*D* = donor in hydrogen bonds, *A* = acceptor in hydrogen bonds) array of quadruple hydrogen bonds. This can be represented by the graph-set notation *R*_2_^2^(8), *R*_4_^2^(8) and *R*_2_^2^(8) (Fig. 2). This type of array has also been identified in trimethoprim hydrogen glutarate (Robert *et al.*, 2001), trimethoprim formate (Umadevi *et al.*, 2002) and 2-amino-4,6-dimethoxypyridinium salicylate (Thanigaimani *et al.*, 2007).  The carboxyl groups of the fumaric acid molecules and the carboxylate groups of the fumarate anions are hydrogen bonded through O2—H2C···O4 hydrogen bonds leading to the formation of a one-dimensional hydrogen-bonded supramolecular chain along the [101] (Fig. 3). This type of carboxyl–carboxylate interaction has been reported in the crystal structures of 2-aminopyridinium–succinate –succinic acid (Büyükgüngör & Odabaşoğlu, 2002) and 2- aminopyridinium–fumarate–fumaric acid (Büyükgüngör *et al.*, 2004). This chain can be designated by graph-set notation *C*_2_^2^(14).  The crystal structure is further stabilized by weak intermolecular C5—H5···O1 (Table 1) hydrogen bonds.

### Experimental

A hot methanol solution (20 ml) of 2-amino-5-methylpyridine (27 mg, Aldrich) and fumaric acid (29 mg, Merck) were mixed and warmed over a heating magnetic stirrer for a few minutes. The resulting solution was allowed to cool slowly at room temperature and crystals of the title compound appeared after a few days.

### Refinement

All hydrogen atoms were positioned geometrically (O—H = 0.82 Å, N—H = 0.86 Å and C—H = 0.93 or 0.96 Å) and were refined using a riding model, with *U*_iso_(H) = 1.2  or 1.5*U*_eq_(C).  A rotating group model was used for the methyl group.

### Crystal data

**Table:**

C_6_H_9_N_2_^+^·0.5C_4_H_4_O_4_^2^^−^·0.5C_4_H_2_O_4_	*Z* = 2
*M**_r_* = 224.22	*F*(000) = 236
Triclinic, *P*1	*D*_x_ = 1.423 Mg m^−^^3^
Hall symbol: -P 1	Mo *K*α radiation, λ = 0.71073 Å
*a* = 4.0366 (4) Å	Cell parameters from 9900 reflections
*b* = 9.3145 (10) Å	θ = 2.7–37.6°
*c* = 14.0077 (14) Å	µ = 0.11 mm^−^^1^
α = 94.030 (3)°	*T* = 100 K
β = 95.060 (3)°	Block, colourless
γ = 90.903 (3)°	0.61 × 0.22 × 0.20 mm
*V* = 523.20 (9) Å^3^	

### Data collection

**Table:**

Bruker APEXII DUO CCD area-detector diffractometer	5445 independent reflections
Radiation source: fine-focus sealed tube	4852 reflections with *I* > 2σ(*I*)
graphite	*R*_int_ = 0.019
φ and ω scans	θ_max_ = 37.7°, θ_min_ = 1.5°
Absorption correction: multi-scan (*SADABS*; Bruker, 2009)	*h* = −6→6
*T*_min_ = 0.935, *T*_max_ = 0.978	*k* = −15→15
19772 measured reflections	*l* = −24→23

### Refinement

**Table:**

Refinement on *F*^2^	Primary atom site location: structure-invariant direct methods
Least-squares matrix: full	Secondary atom site location: difference Fourier map
*R*[*F*^2^ > 2σ(*F*^2^)] = 0.036	Hydrogen site location: inferred from neighbouring sites
*wR*(*F*^2^) = 0.110	H-atom parameters constrained
*S* = 1.05	*w* = 1/[σ^2^(*F*_o_^2^) + (0.0626*P*)^2^ + 0.0878*P*] where *P* = (*F*_o_^2^ + 2*F*_c_^2^)/3
5445 reflections	(Δ/σ)_max_ = 0.001
147 parameters	Δρ_max_ = 0.60 e Å^−^^3^
0 restraints	Δρ_min_ = −0.34 e Å^−^^3^

### Special details

**Table:**

Experimental. The crystal was placed in the cold stream of an Oxford Cryosystems Cobra open-flow nitrogen cryostat (Cosier & Glazer, 1986) operating at 100.0 (1) K.
Geometry. All s.u.'s (except the s.u. in the dihedral angle between two l.s. planes) are estimated using the full covariance matrix. The cell s.u.'s are taken into account individually in the estimation of s.u.'s in distances, angles and torsion angles; correlations between s.u.'s in cell parameters are only used when they are defined by crystal symmetry. An approximate (isotropic) treatment of cell s.u.'s is used for estimating s.u.'s involving l.s. planes.
Refinement. Refinement of F^2^ against ALL reflections. The weighted R-factor wR and goodness of fit S are based on F^2^, conventional R-factors R are based on F, with F set to zero for negative F^2^. The threshold expression of F^2^ > 2σ(F^2^) is used only for calculating R-factors(gt) etc. and is not relevant to the choice of reflections for refinement. R-factors based on F^2^ are statistically about twice as large as those based on F, and R- factors based on ALL data will be even larger.

### Fractional atomic coordinates and isotropic or equivalent isotropic displacement parameters (Å^2^)

**Table:**

	*x*	*y*	*z*	*U*_iso_*/*U*_eq_	
N1	0.64925 (12)	0.45378 (5)	0.20997 (3)	0.01315 (8)	
H1	0.5764	0.3700	0.1870	0.016*	
N2	0.31601 (12)	0.55122 (5)	0.09009 (3)	0.01504 (9)	
H2A	0.2404	0.4662	0.0712	0.018*	
H2B	0.2451	0.6241	0.0605	0.018*	
C1	0.54215 (13)	0.56996 (5)	0.16485 (4)	0.01190 (8)	
C2	0.67623 (13)	0.70674 (5)	0.20110 (4)	0.01341 (9)	
H2	0.6147	0.7890	0.1706	0.016*	
C3	0.89669 (13)	0.71642 (6)	0.28125 (4)	0.01392 (9)	
H3	0.9845	0.8062	0.3048	0.017*	
C4	0.99507 (13)	0.59304 (6)	0.32951 (4)	0.01383 (9)	
C5	0.86767 (13)	0.46307 (6)	0.29027 (4)	0.01433 (9)	
H5	0.9311	0.3795	0.3189	0.017*	
C6	1.21690 (15)	0.60555 (7)	0.42149 (4)	0.01903 (10)	
H6A	1.1001	0.6517	0.4716	0.029*	
H6B	1.4123	0.6617	0.4131	0.029*	
H6C	1.2803	0.5112	0.4388	0.029*	
O1	0.27081 (16)	0.19332 (6)	0.39352 (4)	0.02686 (12)	
O2	0.49662 (14)	0.03168 (5)	0.29314 (3)	0.02170 (10)	
H2C	0.4257	0.0849	0.2522	0.033*	
C7	0.42204 (15)	0.08387 (6)	0.37847 (4)	0.01544 (9)	
C8	0.54504 (15)	−0.00956 (6)	0.45556 (4)	0.01586 (9)	
H8	0.6889	−0.0833	0.4410	0.019*	
O3	0.10076 (14)	0.26911 (5)	0.02053 (3)	0.02176 (10)	
O4	0.34405 (13)	0.19338 (5)	0.15605 (3)	0.02015 (10)	
C9	0.17514 (14)	0.17185 (6)	0.07482 (4)	0.01450 (9)	
C10	0.06922 (15)	0.01973 (6)	0.04396 (4)	0.01504 (9)	
H10	0.1014	−0.0501	0.0880	0.018*	

### Atomic displacement parameters (Å^2^)

**Table:**

	*U*^11^	*U*^22^	*U*^33^	*U*^12^	*U*^13^	*U*^23^
N1	0.01540 (17)	0.00928 (16)	0.01454 (17)	0.00050 (13)	−0.00023 (13)	0.00127 (13)
N2	0.01861 (19)	0.01217 (18)	0.01362 (17)	0.00003 (14)	−0.00253 (14)	0.00090 (14)
C1	0.01360 (18)	0.01021 (18)	0.01198 (18)	0.00080 (14)	0.00146 (14)	0.00086 (14)
C2	0.0165 (2)	0.00966 (18)	0.01402 (19)	0.00031 (15)	0.00102 (15)	0.00067 (14)
C3	0.01491 (19)	0.01202 (19)	0.01459 (19)	−0.00112 (15)	0.00159 (15)	−0.00077 (15)
C4	0.01274 (18)	0.0149 (2)	0.01376 (19)	0.00020 (15)	0.00090 (14)	0.00098 (15)
C5	0.01445 (19)	0.0131 (2)	0.0155 (2)	0.00149 (15)	−0.00004 (15)	0.00273 (15)
C6	0.0157 (2)	0.0245 (3)	0.0163 (2)	−0.00173 (18)	−0.00234 (16)	0.00247 (19)
O1	0.0431 (3)	0.0210 (2)	0.01696 (19)	0.0171 (2)	0.00033 (18)	0.00395 (16)
O2	0.0396 (3)	0.01464 (18)	0.01119 (16)	0.00699 (17)	0.00121 (16)	0.00307 (13)
C7	0.0220 (2)	0.01199 (19)	0.01201 (18)	0.00230 (16)	−0.00174 (16)	0.00234 (15)
C8	0.0221 (2)	0.0134 (2)	0.01226 (19)	0.00444 (17)	−0.00012 (16)	0.00316 (15)
O3	0.0348 (2)	0.01109 (17)	0.01746 (18)	−0.00270 (15)	−0.01071 (16)	0.00485 (14)
O4	0.0338 (2)	0.01254 (17)	0.01229 (16)	−0.00547 (15)	−0.00885 (15)	0.00302 (13)
C9	0.0213 (2)	0.01004 (18)	0.01138 (18)	−0.00190 (15)	−0.00325 (15)	0.00168 (14)
C10	0.0219 (2)	0.01007 (18)	0.01231 (19)	−0.00229 (16)	−0.00368 (15)	0.00215 (14)

### Geometric parameters (Å, °)

**Table:**

N1—C1	1.3492 (7)	C6—H6A	0.9600
N1—C5	1.3635 (7)	C6—H6B	0.9600
N1—H1	0.8600	C6—H6C	0.9600
N2—C1	1.3271 (7)	O1—C7	1.2118 (7)
N2—H2A	0.8600	O2—C7	1.3199 (7)
N2—H2B	0.8600	O2—H2C	0.8200
C1—C2	1.4188 (8)	C7—C8	1.4903 (7)
C2—C3	1.3665 (7)	C8—C8^i^	1.3285 (11)
C2—H2	0.9300	C8—H8	0.9300
C3—C4	1.4184 (8)	O3—C9	1.2468 (7)
C3—H3	0.9300	O4—C9	1.2754 (6)
C4—C5	1.3671 (8)	C9—C10	1.4965 (8)
C4—C6	1.4992 (8)	C10—C10^ii^	1.3314 (10)
C5—H5	0.9300	C10—H10	0.9300
			
C1—N1—C5	123.03 (5)	C4—C5—H5	119.4
C1—N1—H1	118.5	C4—C6—H6A	109.5
C5—N1—H1	118.5	C4—C6—H6B	109.5
C1—N2—H2A	120.0	H6A—C6—H6B	109.5
C1—N2—H2B	120.0	C4—C6—H6C	109.5
H2A—N2—H2B	120.0	H6A—C6—H6C	109.5
N2—C1—N1	118.82 (5)	H6B—C6—H6C	109.5
N2—C1—C2	123.44 (5)	C7—O2—H2C	109.5
N1—C1—C2	117.73 (5)	O1—C7—O2	125.02 (5)
C3—C2—C1	119.37 (5)	O1—C7—C8	123.42 (5)
C3—C2—H2	120.3	O2—C7—C8	111.56 (5)
C1—C2—H2	120.3	C8^i^—C8—C7	121.87 (6)
C2—C3—C4	121.69 (5)	C8^i^—C8—H8	119.1
C2—C3—H3	119.2	C7—C8—H8	119.1
C4—C3—H3	119.2	O3—C9—O4	123.73 (5)
C5—C4—C3	116.88 (5)	O3—C9—C10	119.39 (5)
C5—C4—C6	121.66 (5)	O4—C9—C10	116.87 (4)
C3—C4—C6	121.41 (5)	C10^ii^—C10—C9	122.67 (6)
N1—C5—C4	121.22 (5)	C10^ii^—C10—H10	118.7
N1—C5—H5	119.4	C9—C10—H10	118.7
			
C5—N1—C1—N2	−176.50 (5)	C1—N1—C5—C4	−0.74 (8)
C5—N1—C1—C2	2.78 (8)	C3—C4—C5—N1	−1.74 (8)
N2—C1—C2—C3	176.95 (5)	C6—C4—C5—N1	175.74 (5)
N1—C1—C2—C3	−2.29 (8)	O1—C7—C8—C8^i^	10.08 (12)
C1—C2—C3—C4	−0.13 (8)	O2—C7—C8—C8^i^	−169.47 (8)
C2—C3—C4—C5	2.14 (8)	O3—C9—C10—C10^ii^	7.89 (11)
C2—C3—C4—C6	−175.35 (5)	O4—C9—C10—C10^ii^	−170.95 (8)

Symmetry codes: (i) −*x*+1, −*y*, −*z*+1; (ii) −*x*, −*y*, −*z*.

### Hydrogen-bond geometry (Å, °)

**Table:**

*D*—H···*A*	*D*—H	H···*A*	*D*···*A*	*D*—H···*A*
N1—H1···O4	0.86	1.89	2.7305 (7)	167
N2—H2A···O3	0.86	1.98	2.8334 (7)	175
N2—H2B···O3^iii^	0.86	2.04	2.8329 (7)	154
O2—H2C···O4	0.82	1.75	2.5618 (7)	170
C5—H5···O1^iv^	0.93	2.46	3.3582 (9)	162

Symmetry codes: (iii) −*x*, −*y*+1, −*z*; (iv) *x*+1, *y*, *z*.
